# The Epic of In Vitro Meat Production—A Fiction into Reality

**DOI:** 10.3390/foods10061395

**Published:** 2021-06-16

**Authors:** Balamuralikrishnan Balasubramanian, Wenchao Liu, Karthika Pushparaj, Sungkwon Park

**Affiliations:** 1 Department of Food Science and Biotechnology, College of Life Science, Sejong University, Seoul 05006, Korea; geneticsmurali@gmail.com; 2 Department of Animal Science, College of Coastal Agricultural Sciences, Guangdong Ocean University, Zhanjiang 524088, China; liuwc@gdou.edu.cn; 3 Department of Zoology, School of Biosciences, Avinashilingam Institute for Home Science and Higher Education for Women, Coimbatore 641 043, Tamil Nadu, India; karthika_zoo@avinuty.ac.in

**Keywords:** cultured meat, stem cells, meat substitute, eco-friendly, technical challenges, acceptance, 3D bioprinting, nanosensors, multidisciplinary approaches

## Abstract

Due to a proportionally increasing population and food demands, the food industry has come up with wide innovations, opportunities, and possibilities to manufacture meat under in vitro conditions. The amalgamation of cell culture and tissue engineering has been the base idea for the development of the synthetic meat, and this has been proposed to be a pivotal study for a futuristic muscle development program in the medical field. With improved microbial and chemical advancements, in vitro meat matched the conventional meat and is proposed to be eco-friendly, healthy, nutrient rich, and ethical. Despite the success, there are several challenges associated with the utilization of materials in synthetic meat manufacture, which demands regulatory and safety assessment systems to manage the risks associated with the production of cultured meat. The role of 3D bioprinting meat analogues enables a better nutritional profile and sensorial values. The integration of nanosensors in the bioprocess of culture meat eased the quality assessment throughout the food supply chain and management. Multidisciplinary approaches such as mathematical modelling, computer fluid dynamics, and biophotonics coupled with tissue engineering will be promising aspects to envisage the future prospective of this technology and make it available to the public at economically feasible rates.

## 1. Introduction

Dozens of small-scale and large-scale firms are functioning to develop cultivated poultry, sheep, beef, and pork that escalate the industrial livestock production for providing cleaner, residue-free, and cruelty-free meat [1]. Surveys estimated that two-thirds of the mammals on earth are livestock, one-third constitutes humans, and the remaining feeble numbers account for wild animals [2]. Meat and meat products are the most common and most widely consumed foods. They form the essential and expensive source of animal protein; the consumption of meat has increased exponentially due to the growing population. It is predicted that by 2050, intake of meat will be magnified several times, so that conventional meat supply will not be sufficient to meet the demands [3]. Complementary approaches such as in vitro meat production is one of the upcoming alternative sources to match the meat supply demands.

Since the debut of the first cultured meat burger patty in 2013, several multinational concerns have invested to commercialize lab-grown meat products. This technique involves meat production through tissue-engineering technologies and cell culture methods without engaging with animal rearing and slaughtering. This type of meat production under controlled laboratory conditions facilitates health, animal welfare, global environmental conditions, and financial systems as well [4]. Traditional meat production systems involve the rearing of ruminants, which are accountable for 37% of methane release. Overgrazing of lands and deforestation also added up to the increase in greenhouse gases [5]. There are several other moral issues such as unethical slaughtering; religious practices are also bound with the consumption of real meat. The intake of meat also added up to several health-related complications such as cholesterol-related disorders, cardiovascular disease, endoparasitic infections, and food-borne infections caused by microorganisms. The call for demand is high, which requires immediate alternative strategies for meat production and smooth global food supply chain. Therefore, a pressing priority is ensuring protection from the emerging infectious diseases from livestock as well as to minimize overgrazing for rising domestic flock for food and also to ensure animal welfare. In view of the above problems, artificial meat seems to be a potential alternative source for conventional meat and is much welcomed by the food technologists. Generally, artificial meat is manufactured from three basic types of natural substitutes from non-animal sources, plants, or fungus [6]. Soya meat is engineered with plant-based proteins [7], laboratory-cultured meat, or in vitro meat from the cell lines and others from the genetically modified organisms such as transgenic pigs and cows for the production of cheese and milk [8] and even Enviropig [9] for the production of omega 3 fatty acids. Inventions and discoveries of the current era have made it possible to obtain meat cultured from the seed muscle tissue taken from live animals’ biopsies or animal embryos, enriched in a medium, and grown in a bioreactor under controlled conditions [10].

In spite of these strong interventions, large-scale or commercial production of unprocessed meat warrants deep knowledge on the concept of research and high-end research facilities, which directly reflects on the extremely high cost at the consumer end. However, in the long-term process, in vitro meat will inevitably become food for human consumption in future. The satisfaction of food for the growing population can be brought about by the supply of in vitro, artificial, or lab-grown meat and is also promoted as a forceful alternative for consumers who do not want to alter the non-vegan diet [5]. The present review outlined the existing knowledge, inventions, processing methods, issues, and ethics pertaining to the cultured meat. It also emphasizes recent biomedical and technological interventions bridged with the food industry for the production of the same. However, no advances were recorded in spite of research publications [11,12,13,14,15]. It requires more integrated input and novel techniques in cell culture protocols that enhance rapid and mass production and also availability at cheaper rates to ensure customer satisfaction. The reproduction of different types of meat, i.e., diversified species, breeds, etc., may not be possible, and speculation on the nutritional benefits needs to be clarified.

## 2. Scope and Key Findings

The prospects of lab-grown meat address the facets of food longevity and nutritional security and are posed to be a potential complementary approach for conventional meat. The feature that in vitro meat is manmade has created a happy deal for animal lovers and, in parallel, has circumvented the chances of several zoonotic diseases and human–animal interactions. The greater part of the success of in vitro meat laid in the production of pathogen-free, environmentally friendly meat, which can reduce the methane emissions from conventional animal farming. However, challenges such as socio-economic and technological interventions play a decisive role in the availability of in vitro meat to common people at affordable rates globally. The review covered the bright side and challenges in cultured meat technology. It also highlighted on the aim of bringing the meat from Petri dish to the plates of consumers with the innate taste and flavor of original meat. The governing factors behind survivability and viability of this technology have been underpinned. Further, the cost and production effects of this meat technology have also been discussed. Public acceptance, infrastructure inadequacy, and financial management were the governing factors that promote cultured meat in the global market. The recent pandemic has also provoked the consequence of human–animal interactions and also underlined the basic need for muscle meat during lockdowns. However, a thorough substitution of conventional meat with in vitro meat may be partially disagreeable in agro-based countries, but cultured meat can be an irresistible complementary approach in developing countries to quench the food needs of the human population. In this review, we discussed the crosstalk between cell culture and technology and the supporting factors in the development of in vitro meat. This review also emphasizes the critical role of the academic sector in making the public understand the actual science of cultured meat and bringing awareness of its environmental benefits into the limelight. Moreover, acceptance of this lab-tailored food by all religious communities was the most welcoming aspect. However, a clarification of perceptions needs to be addressed through research, and the implementation of potential policies will help to achieve maximum benefits for planetary health.

## 3. The Timeline and Episodes of In Vitro Meat

The history of cultured meat involves a combination of surreal predictions and scientific findings as well. The pilot idea was put forth by Alexis Carrel, who cultured the steak of chick heart muscle in a Petri dish under live condition. In 1930, Frederick Edwin Smith, a politician, predicted that it would not be necessary to wait for a long period of time to eat buffalo fillet [16,17]. Winston Churchill in 1932 mentioned the idea of in vitro meat in the essay entitled “Fifty Years Hence”, which was then published in the book *Thoughts and Adventures*. Interestingly, Rene Barjavel, a French science fiction writer, mentioned in vitro meat in restaurants in his novel in 1943. In 1953, Willem Van Eelen of The Netherlands promoted the culture of meat based on tissue engineering. While, NASA, in collaboration with Benjaminson and his research team of Germany, cultured the muscle tissue from *Carassius auratus* in petri dishes and supplied them as food to astronauts after the ethical approval of panel members in 1997 [16]. During the year 1999, the renowned laboratory (SymbioticA) harvested muscle biopsy from frogs and engineered in vitro cells [17], while Van Eelen patented the theoretical idea of the same. In 2003, Mark Post, a scientist, cultured meat in his laboratory at Maastricht University, The Netherlands. The revolutionary progress was recorded in the year 2013 when the first in vitro meat-based burger was prepared and presented to the panel by the Riverside Studios of London. Later, in 2013, New Harvest invited the start-up schemes to enhance the cellular agriculture for in vitro meat production. Several cultured meat projects such as Shojin Meat Project, Memphis Meat, Super Meat, and Finless Foods were initiated in 2014, 2015, 2016, and 2017, respectively. Finally, a US-based start-up, Eat, debuted the in vitro chicken meat in the restaurants of Singapore in December 2020, and it was the first country to approve the sale of cultured meat. According to the recent review reports in 2020, the initial literature pertaining to cultured meat contained fourteen studies spanning around 2014–2018, while the publication numbers doubled after 2018, totaling twenty-six empirical studies based on consumer acceptance of cultured meat. Notably, most of the research/reviews/commentaries were published in peer-reviewed journals. In parallel, advancements in the domain of tissue culture technology and integration of multi-disciplinary approaches still continue to flourish in grey literature [18].

## 4. Animal Welfare

Amongst wide benefits of in vitro meat, some of the prominent advantages include the reduction in the suffering of the livestock, elimination of foodborne and nutrition-related diseases, and minimized greenhouse gas emissions [12]. Laws and legislations exist globally for the animal sentence, which debate on their capacity to withstand suffering and pain, and this has been legally recognized by European Union laws. Hitherto, the laws that inflicted unnecessary suffering upon the livestock maintained under human care have been prohibited and considered morally indefensible, and the act of suffering should not be neglected [13]. The suffering of animals in factory and livestock farms are closely associated with animal welfare. Animal farming is dependent upon factors such as market value, land, and sustained resources; inadequate availability of these factors can reduce the wellbeing and heath of the reared animals [14]. However, such reared animals are categorized for slaughtering; hence, not much significance is given to their health, and they are susceptible to nutrient deficiency diseases. Ultimately, the animal welfare measures are reduced to a minimum or ignored in livestock farms. Poor ventilation and over crowdedness leads to chronic illness and induced stress throughout their lifetime [15]. Even with stringent guidelines proposed by international governments, deprived legal protection is ensured; however, at the national level, these are poorly enforced [19]. Evidence on the routine killing of animals without proper protocols such as traumatic mutilations, amputations, and castrations without anesthesia have been recorded [14].

Given these options, cultured meat has the greatest potential to eliminate the pain and suffering of animals during slaughter. Proportionally, the number of animals required for cell culture is much smaller in magnitude compared to the whole animals required for conventional meat [20]. Thus, a single “parent cell” obtained from the explant serves for the production of meat for nearly a year. This has set a benchmark for the production of meat globally in laboratories. The chief aim of the process is the recreation of the multifaceted muscle tissues from a few cells that were sampled from a biopsy procedure from a live animal. The appreciable property depends on the proliferation of these cells into different types of cells (e.g., muscle cells, fat cells, etc.) [21]. The leap of in vitro meat circumvents the moral and ethical ramifications associated with traditional meat harvest by escaping cruelty and animal death [22]. Hence, cultured meat is often referred to as victim-less meat, and it is most welcomed by animal activists and non-vegan nutritional experts. Experts envisage that an improvement in technology for the culture of embryonic stem cells, which are innately bound with a self-renewal capacity, will underpin the meat industry [23]. The maintenance of fewer cell lines will be sufficient to produce feed for the entire world, with an approximate yield of 50,000 metric tons of meat [24].

## 5. Choice of Cell Lines for In Vitro Meat Production

The pioneer proposals were put forth by Vladimir Mironov for the NASA [25] and Willem van Eelen [26] based on tissue engineering and cell culture techniques that ventured into in vitro meat production [27]. The baseline idea for the proposals relied on the utilization of collagen spheres for adherence and collagen meshwork with periodical replacement of the medium for the culture of myoblasts. Recently, microstructure edible films were used to grow muscle fibers [28]. As of today, science is trending with novel branches of biotechnology such as biomaterials, cellular engineering, and cellular agriculture to address issues related to environmental, animal, and human welfare. In the context of in vitro meat, the utilization of cell lines and the biomanufacturing process have played a vital role in the tissue generation coupled with nutritional proteins for consumption [29]. According to this study, commercial production is ensured without antibiotic residues and minimalistic chances of foodborne illnesses due to enteric pathogens.

**Figure 1:**
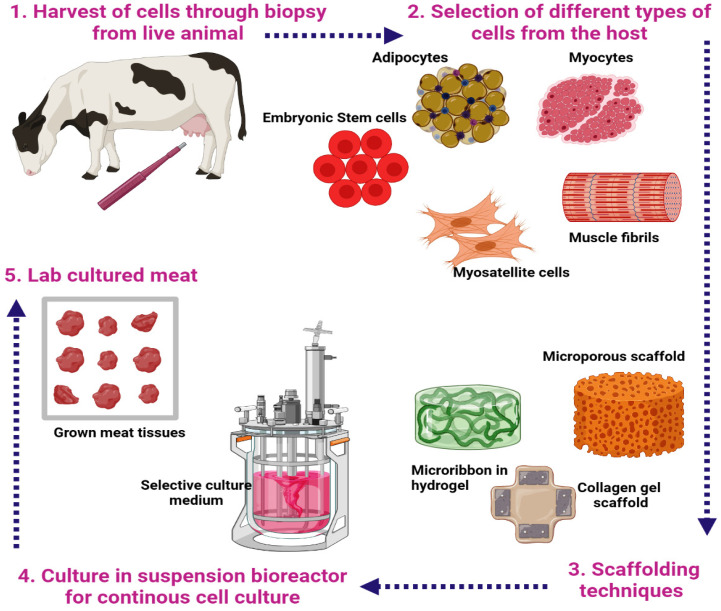
A pipeline for the production of illustrating the common stages involved in the production of a cultured meat product.

Several choices of cell lines are open for culturing meat, but refined research is needed to precisely determine the properties of cell lines that directly influence the downstream regulation process. Figure 1 illustrates the pipeline of the in vitro meat culture. Common types of stem cells such as muscle cells [30], satellite cells [31], adipogenic stem cells, and induced pluripotent stem cells [32] are the best candidates. The selection of cell type is personalized; for example, meat is a protein-rich material, to complement the protein composition in the cultured meat, muscle cells that produce tissues that are innately rich in protein are used, while satellite cells possess improved regenerative capacity [31]. In parallel, stem cells have the remarkable uniqueness of being undifferentiated and are identified as the best-suited candidates for in vitro meat culture. On the other hand, induced pluripotent stem cells differentiate into myotubes facilitating in vitro muscle repair. Another choice of utilizing adult skeletal muscle cells has an advantage for the production of cytoskeletal proteins, which are the best protein sources one could benefit from when cultivating cultured meat. Apart from the basic choice of cells, specialized types of cells such as endothelial cells, which hold the property of proliferation and differentiation of muscle progenitor cells to tissues, are employed [33], and these are also reported to influence adipogenesis [34]. In addition, an extracellular matrix secreted by microvascular endothelial cells and fibroblasts kindle preadipocyte differentiation and muscle maturation, which enhance the texture of meat [35].

In addition, multipotent cells of adipose tissue called adipose tissue-derived adult stem cells (ADSCs) were found suitable for in vitro meat production [36]. These cells were identified and isolated from the subcutaneous fat and had the potency to consequently transdifferentiate into varied cell types such as myogenic, osteogenic, chondrogenic, or adipogenic cell lineages [37]. In parallel, the cell ceiling method was developed based on the dedifferentiated fat (DFAT) cells that originated from mature adipocytes [38]. These cell lines are exclusively capable of transdifferentiating into skeletal myocytes and serve as a potential alternative for naive stem cells [39]. In addition, fresh stem cells obtained through biopsy from live animals from any organs such as mammary glands and liver can be employed for culture in specific mediums in bioreactors. In compliance to the religious laws (e.g., Halal, Kosher), stem cells can be harvested from recently slaughtered animals, provided the tissues are be viable. However, the major challenge associated with the cell lines is the self-renewal and uninterrupted proliferative capacity. Mimicking conventional meat is truly challenging, and it typically involves tissue-engineering protocols to provide a scaffold to support the organization of the cells and assemblage of tissues that closely match in the nutrition and flavor of real meat [40].

The choice of cell lines, however, also depends on the option of producing single muscle protein or complex tissues comprising fat, nerves, blood, and immune cells as well. Nevertheless, the assemblage of nutrients and the cytoskeleton architecture prompt the success of the cultured meat. Alternatively, in clinical and medical research, tailoring new cell lines requires proportionally huge funds, long time periods, and large-scale cell line biorepositories, which can usually be sponsored by national governments. An intervention of genetic engineering provides a loop for inducing desired changes, while this becomes an option for the meat production companies to patent their techniques. In spite of all these innovations and novel techniques, regulatory ethical approval determines the final form of product at the customer end.

## 6. Reduction in Zoonotic Diseases

The global population is predicted to reach 9.7 billion in 2050 [41]. Due to the influence of global megatrends, there is a notable drift of people changing from vegan to non-vegan foods, which has become increasingly important. In the process of globalization, factors such as economic development and urbanization have influenced the eating habits of middle-class groups to an affluent menu [42]. Surveys suggested that populations in Asian countries have shifted over to meat and animal products from plant-based foods, provoking the global demand of meat products, which is expected to exponentially increase up to 73% in 2050 [43,44]. 

Recent pandemic outbreaks such as COVID-19 have invited society to revamp on risk prevention policies of infectious disease. Mackenzie and Smith [45] reported that COVID-19 was the only candidate with pandemic potential, while the previous infection caused by SARS-CoV and MERS-CoV had less virulence. Recent studies have suggested that 75% of the infectious diseases are of zoonotic origin [46,47] and are associated with raw meat consumption. Meanwhile, animal farming and wild meat markets play crucial roles in the outbreak and amplification of infectious diseases [48]. Mitigation strategies are successful only with the proper identification of the problem. Hence, to avoid the new emergence of infections and to sustain a healthy life, alternatives for meat become the need of the hour. Replacement of traditional food systems such as bushmeat [49] and backyard farming [50] will lower the risks, while cut-down plans of intensive farming will reduce the risk of disease amplification [51]. Moreover, the chances associated with the exponential spread of infection can be lowered by banning the live transport of animals [52] for slaughtering across boundaries. All these plans will undoubtedly reduce the impact of disease through the feeble density [53] and decreased genetic proximity [54] of farmed animals [55].

A plethora of scientific evidence suggested that most virulent zoonotic diseases are caused by the consumption of raw meat, which is a result of human–wildlife interactions [56]. Examples of evidence are quoted from several countries such as Botswana, Ghana, Cameroon [57], and China [58], while the prominent examples include Ebola (bat) [56] HIV (chimpanzees), anthrax (ungulates), and Simian foamy viruses (gorilla) [59]. The implementation of regulatory frameworks [60] and emergency preparedness [61], particularly innovations such as laboratory-cultured meat will definitely improve the biosecurity of the stakeholders [47]. The promise of cultured meat will permit the production of meat without the pipeline of rearing, transport, and butchering but with the exquisite flavor and uncompromised taste [62]. In view of these facts, cultured meat is free from infections and food-borne pathogens, which drastically reduce the risks of zoonotic disease [63].

## 7. Top Five Reasons for the Need of Cultured Meat

### 7.1. Option of Customizing the Nutrient Profile in In Vitro Meat

The success of in vitro meat culture depends on the choice of the culture medium. Moreover, the selection of culture manipulates and influences the flavor and fatty acid composition so that the raw cultured meat can be customized according to the nutritional requirements. These are often referred to as designed meat, which has the extra advantage of health benefits due to the inclusion of certain types of vitamins [11]. For instance, the customization of fat-enhanced meat is carried out with the co-culture of fat-producing adipocytes. This phenomenon complementing meat with additional flavors is welcomed at the customer end, while the advents of the nutritionally altered foods have attracted stakeholders’ willingness towards specific nutritional characteristics [64]. Similar to conventional meat, the designer meat does not offend the sentiments and emotions of animal lovers and is preferred, as it is safer, pathogen-free, eco-friendly, and ethical, which ensures the satisfaction of all food lovers [11].

### 7.2. Minimalistic Utilization of Bioresources and Improvement of Ecological Footprint

The alarming projection of the increase in greenhouse gases is threatening, while a major part constituting 7.1 gigatons of carbon dioxide emissions accounting for about 14.5% of emissions [65] is sourced from livestock supply chains [66]. Rearing livestock for meat and dairy products occupies 65% of the total greenhouse gas emissions; particularly, 45% of these are in the form of methane [67]. This rapid variation of climate change directly imposes fluctuations in wildlife distribution and accelerates the spread of zoonotic and vector-induced infectious diseases in humans and wildlife [68]. Meat production through livestock rearing has greater association with freshwater consumption, while it is estimated that one-third of freshwater resources are utilized for animal farming [69]. Water used for growing animal feed accounts for 98% of the total water footprint of livestock production [70]. However, there is a notable variation in the water footprint based on meat production through plant-based products and synthetic diet, which are given as feed for livestock. Therefore, a significant reduction in the carbon footprint in reality is possible with the production of meat in a laboratory, which undoubtedly ensures the ecological balance.

### 7.3. Religious Taboos and Acceptance of Cultured Meat

**Figure 2:**
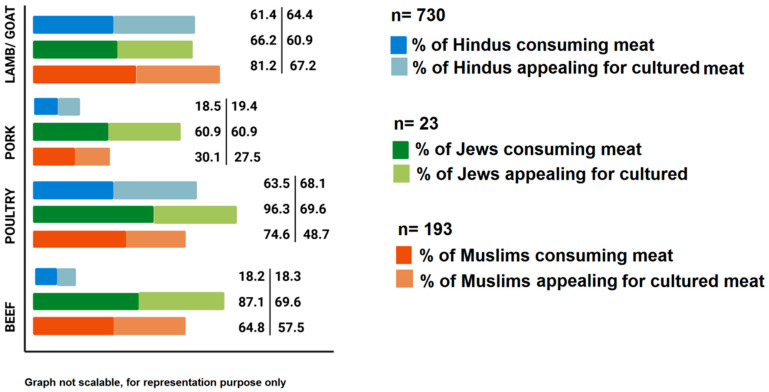
An illustration of the acceptance of in vitro meat across the three religions [18].

Recent surveys on the acceptance of cultured meat across several countries involving Australia, China, England, France, Germany, Mexico, South Africa, Spain, Sweden, and the US suggested that there was a large cultural difference associated with it [71]. The demographic profile of meat consumers based on religion constitutes 1.8 billion Muslims, 1.1 billion Hindus, 0.5 billion Buddhists, and 10 million Jews [72]. In vitro meat tailored from live animal cells has the challenge and prospects to convince several social–ethical, eco-friendly, and public health issues. Yet, overcoming and advocating for sensitive issues such as religious acceptance remains a great task [18]. Figure 2 illustrates the percentage of three religious populations (Hinduism, Islam, and Judaism) having appeal towards artificially grown meat. Convincingly, laboratory-engineered meat does not come from a present live animal, and it, therefore, abides by the religious laws such as Jhatka, Halal, etc. [10]. Judaism and Islam unanimously approved that cultured meat is Kosher [73] and Halal [74], respectively, provided the cells of the slaughtered animal are harvested in an ethical way. However, Hinduism and Buddhism have several principles of non-violence, which encourage a vegan diet, and a feeble percent of Hindus prefer cultured meat as most encourage the harmless killing of animals for food [75]. However, the consumption of beef is prohibited, as they consider cows to be sacred.

### 7.4. The “Future Food”

Unlike conventional meat production, laboratory-grown meat requires less time and produces maximum yield, so that one single animal is enough to feed the population. Moreover, the traditional pipeline for livestock requires the exhaustion of energy and bioresources and the natural needs such as fodder, locomotion, and space, and reproduction requires the stipulated time and investment of money. Contrastingly, this designer meat has the advantage of being produced in bioreactors that can be a step up in much less space, and the harvest can be rapidly done with all possible customizable nutrients such as vitamins, proteins, minerals, and add on nutrients as well. The strategic aim of these bio-invented foods has much significance, as they can be carried easily to supply troop encampments in warfronts in polar and tough terrain areas, where the supply of nutritional food becomes a difficult task. In the same way, these can be provided as food to astronauts where a wholesome and ample amount of energy has been provided to them. Additionally, these have been used as foods to scientific stations camped in oceans and high latitudes where protein and fat-rich foods become a mandatory need. Thus, the in vitro meat has a prominent advantage in satisfying the food quest in emergency situations and also fulfils long-term survival [76].

### 7.5. Rejuvenation of Forest Cover and Legal Feasibility of Exotic Meat

**Figure 3:**
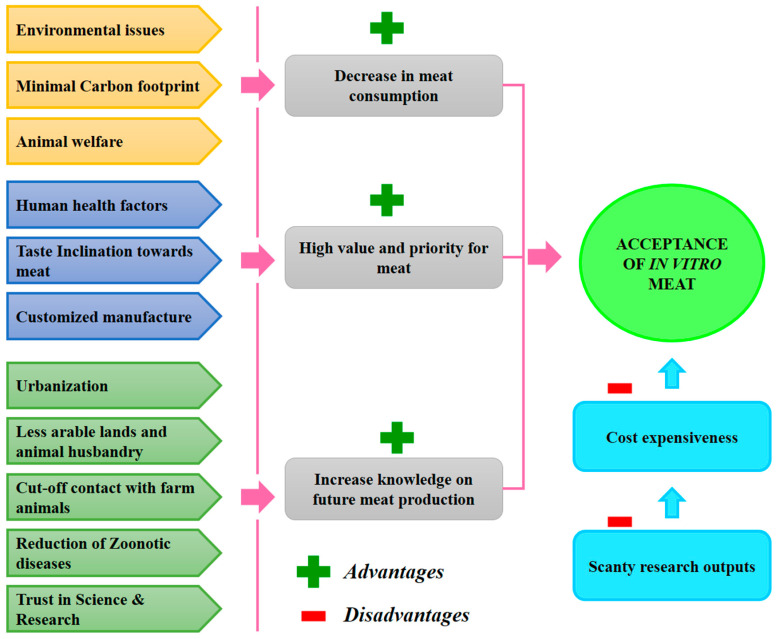
The agenda underpinning the promotion and acceptance of artificial meat.

The utilization of forest covers for grazing and fuel are directly linked with livestock rearing for meat production. The promotion of in vitro meat can certainly influence the reduction in the exhaustion of forest resources and support environmental sustainability for the long term. Moreover, it also becomes a great way of restoring the endangered animals pertaining to their ecosystem and intensely promotes the hotspot quality of a nation. In other words, this technology can prevent wildlife poaching activities for the exotic meat of animals. Since cells can be cultured in a lab using stem cells, the culture of exotic meat is also possible. This directly reduces the threats to wild animals from being extinct. It is reported that the conventional global marketing of rare and endangered animal meat is alarming and has reduced the wild populations of many rare species in many countries [62]. Figure 3 explains the overall governing factors and the hurdles for the acceptance of cultured meat.

## 8. Nanotechnology-Based Approaches for Cultured Meat Production

Muscle foods such as meat are rich sources of high-quality macronutrients such as proteins, essential amino acids and multivitamins, fat and essential fatty acids, minerals, and other micronutrients [77]. Muscle foods are rapidly perishable leading to a reduction in nutrient quality and food spoilage. Alternative approaches such as gene editing, selective breeding, and modern nanotechnology are adopted to fortify the muscle foods with the required nutraceuticals and preservatives for a long shelf life without nutrient reduction [78]. In the given way, Das et al. [79] reviewed the formulation and fabrication of food-grade nanoemulsions and their potential benefits and limitations in muscle food systems. Healthy vigor and breed selection underpin the success of animal breeding. A recent study benefitted the selection of viable sperms through magnetic nanoselection via high-throughput targeting approach. This phenomenon of using magnetic nanoselection for sorting and removal of abnormal spermatozoa from semen tend to be a promising tool for improving male fertility [80]. While researchers are working underway to bring advancement in nanotechnology through nanorobots, which are inbuilt with the capacity to assort molecules for providing the structural framework [81,82] in case of culture meat.

The integration of biophotonics with nanotechnology provided better anchorage of cultured cells, which improved coax in an organized and uniform manner. This can be an alternative method for holding cells in position instead of adopting conventional scaffold techniques [83]. Smart packaging systems have revolutionized the food packing industry by providing authenticity and monitoring the food product during storage and transit as well. The incorporation of nanodevices along the food items provides the facility for genuine tracking and also to check the expiry date of the products. Moreover, it can also provide safe standards against counterfeit products [84].

The detection of meat spoilage is of great concern; remarkable advancements of food based nanosensors such as e-nose, e-tongue, lab-on-chip nanosensors for pathogen detection, surface-enhanced Raman scattering-based sensors, and aptamer-based sensors are exclusively applied to identify the presence of microbial pathogens, contamination, and toxins as well. They offer potential advantages such as rapid sensitivity, accuracy, and functional detection to remove the contaminants and aid in providing quality meat to the stakeholders [85]. In vitro meat is often termed as designer meat due to the customization of the nutrients; this idea can be formulated further by the utilization of nanotechnology-based methods. Meat fortification can be achieved by the intrusion of bioactive or functionally required compounds through application of nanodelivery systems such as nanosuspensions, nanoemulsions, nanoliposomes, and cyclodextrin carriers, which can overcome the drawbacks of unsatisfactory taste/flavor profiles, reduced stability, and bioavailability [86]. The role of nanotechnology in meat packaging has quite a lot of advantages such as mechanical tolerance, heat-resistant properties, enhanced biodegradability, and improved barrier properties. They can also be used as packaging materials, which are coupled with anti-microbial enhancers and spoilage detectors. While nanolaminates or edible coatings can be used in the wrapping of the meat and other food products through layer-by-layer (LbL) deposition techniques, which can extend the shelf-life of the products. These innovative packaging systems ascertain the meat quality during transit, which enables preservation and potential distribution and also aids in the smooth communication pipeline at the consumer levels [87]. Very recently, an integrated technology-based critique, “embodied multi-material layering of in vitro meat”, was developed to study state-of-the-art technologies that collaborated bioinformatics with the agri-food sector and biomedical engineering [88].

## 9. 3D Bioprinting of Meat Analogues

The era has been pivotal in developing innovations that are multifaceted, technologically feasible, environmentally sustainable, and customer friendly. This promising technology is identified to have an unparalleled potential to fabricate meat and other food products at a reduced rate, minimal energy, reduced CO_2_ emissions, and lower lifecycle energy demands for manufacturing goods [89]. The fabrication of meat analogues using 3D-printing (3DP)-based approaches has been one of the cutting-edge technologies of this decade. The process of 3DP involves the deposition of materials or inks in a LbL fashion to form the intricate 3D structures [90]. Biofabrication using 3DP is based on the materials similar to laboratory-cultured muscle cells (in vitro meat), by-products or meat waste, insect proteins, and plant products. However, the challenges include lack of food-safe substrates/materials, cost effectiveness, and scalability. The robustness of bioprinting with meat analogues could be improved by scaffolds and the optimization of cell cultures and fabrication logistics.

The most commonly used 3DP methods include extrusion, inkjet printing, binder jetting, and bioprinting [91]; however, meat fabrication is typically done by the extrusion method due to the presence of coarse muscle fibers and the tough semi solid consistency of the meat puree that is used as the raw material [92]. The 3D bioprinting uses extrusion-type printers and various 3D models employed for creating the structural meat variants [90]. Cultured meat mimics of specific portions such as beef steak, pork shoulders, etc., often requiring spatial resolution and redefinition due to the presence of vasculature and intramuscular fat, which will impact on the taste, flavor, and texture to mimic the original ones. This can be resolved through 3DP through improving scaffolds (preferably hydrogel scaffolds), introducing multiple muscle types, and even producing functional organs in ex vivo conditions [93]. Tailoring vasculature improves the volume in cultured meat; however, it is challenging due to limited diffusion among the cultured cells and the media [94]. In parallel, initiation of a complex tissue structure through a culture of multiple cell-types can be overlooked by creating connective tissue structures by an edible matrix (scaffolds) made of collagen and elastin [95].

**Figure 4:**
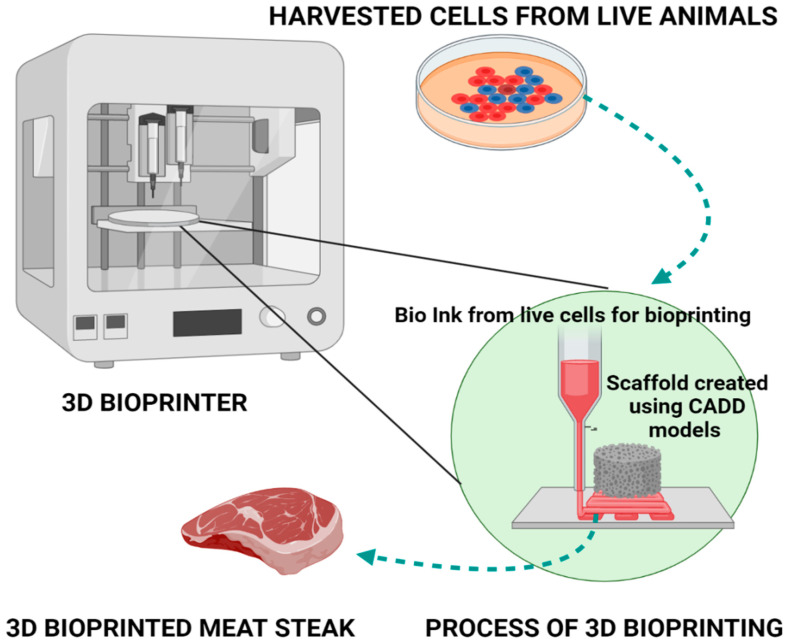
The trans-disciplinary application of 3D bioprinting in in vitro culture meat technique.

The 3D printing of muscle is characterized by cell distribution and alignment and synchronization in the contraction; however, critical challenges include the biochemical compatibility, resolution, and throughput [96]. A recent study reported that the choice of bioprinter nozzle, pressure, and shear stress influence the growth and differentiation of the cells in the case of mouse myoblast cell culture (C2C12); in addition, the mechanical properties of “meat-ink” are also a determining factor of the 3D-printable meat products [97]. Overall, the advantages include the speed of production, customization of forms, homogenous distribution of nutritional content (protein, fat) [98], and easy of handling even in space stations [99]. In 2021, an Israeli concern, “Aleph farms” for the first time produced the 3D bioprinted rib-eye steak for the first time [100]. Figure 4 illustrates the incorporation of 3D bioprinting with in vitro meat technology.

Nevertheless, 3D printing could provide distinctive solutions for regulating the nutrient profile of the cultured tissues, particularly fat and proteins, the vital issues of cultured meat production, especially on regulating the protein, fat, and other nutritional content, along with providing realistic texture [97]. In future, 3DP holds realistic potential in the food sector to tailor 3D foods with selective nutrient and custom-made texture and geometry as well. However, a crucial issue concerned with the acceptance of 3DP foods relies on their unusual appearance. While, several studies claim that these fabricated foods should be considered as novel to promote business and development for a more sustainable food chain in future. Interestingly, the experts envisage the future standpoint would be a culmination of 3DP combined with cooking on a single device, which is a basic prospective for the development of 4D printing devices to facilitate the food chain supply and management [101]. So, what to expect in next ten years? This technology in association with the meat industry can bring about the industrial revolution through the utilization of food wastes such as meat-cut offs, etc., to create a wholesome palatable platter of meat, sea foods, or any vegan foods with the close texture and taste of the respective natural food. The combination of in vitro meat and 3DP technology offers an uncompromised solution for eliminating the future food crisis, avoids animal cruelty, and completely reduces GHG emissions and water wastage. The infusion of nutrigenomics can effectively produce personalized food based on an individual’s genetic information, lifestyle disorders, and deficiency disorders [102].

## 10. Challenges and Awareness of Cultured Meat

Psychological acceptance among the stake holders formed the basis of saying “yes” to the cultured meat products. The “awareness” of cultured meat is the finest predictor of acceptance. Consumer acceptance faces major hurdles, in spite of cultured meat being an undebatable option for environmental safety; the negative perceptions are seen as a major challenge. To the point, if no one consumes the cultured meat, there is no possibility to benefit the environment in this regard. Research claims that this is common among humans and the term is as neophobia, a state of predisposition to unfamiliar or uncommon foods. This barrier can be resolved through awareness, transparent discussions on public forums, and research outputs by the academic people of scientific background to the public to resolve the skepticism. The preconceived notions should be clarified, and necessary proactive strategies are required for the promotion and increased acceptability of the in vitro meat [103]. The media plays a vital role in promotion as well as critics for any new technology and is subject to debate of or acceptance. Buyer approval of cultured meat is likely based on broad determinants such as knowledge, perceptions, product expectations, and other factors such as policy decisions, trust in science, public advertisements, and media coverage [104].

On the contrary, a moral opposition is put forth from the farmers and livestock growers that this technology functioning as part of cellular agriculture will grab away their livelihood [105]. Cultured meat aims to provide newer opportunities to conventional agriculture by the maintenance of traditional native breeds of livestock. The transit from slaughtering carcass to cell harvesting underpinned the selection of high-yielding breeds based on the genomic and phenotypic traits for conventional rearing to the livestock, which can strive with low input and extensive set up. This can give a three-fold rise in the benefits, including reduced environmental impacts, conservation of biodiversity in terms of indigenous breeds, and high marginal profit. The chief consideration comes from the context of waste management in carcass utilization in the meat industry, more quantity of wastes are generated, while a prime cut is optimum for the process of cultured meat than handling the whole carcass. Nurturing the skill of the producers and upgrading their field expertise will take this industry to the next level and become competitive as well. It can generate various job opportunities, and a combination of traditional animal farming along with cutting-edge technologies ensures a circular economy, as the excess energy and metabolites generated during culture can be re-used in the farm [106].

Much research has been pivotal in taking this technology into the purview of the common public. As evidence in reality, Rolland and his team [107] recorded positive responses and acceptances from a group of volunteers in The Netherlands who were offered to eat the conventional and cultured hamburgers. Similarly, the European markets also welcomed cultured meat, while the prominent acceptance was recorded from the agricultural and meat workers who preferred to accept this as a best alternative for traditional meat. On the other hand, the German and French consumers liked the reduced antibiotics and genetically unaltered conditions in the cultured meat. These highlight the promising aspects of acceptance in the European markets [108]. Initiation by policy makers and support through government funds and marketing campaigns will prompt the emergence of start-ups and will provide concomitant exposure to society and play a prominent role in influencing the public’s perceptions of cultured meat [4]. A primary exploratory study conducted in Belgium with 180 participants revealed that most of the participants expressed a liking towards the cultured meat, while 9% objected, 43% indicated willingness, and 51% were hypothetical on accepting it. However, vegans were not convinced, and similar information was also reported in other surveys; hence, this particular group may not be the ideal target for approaching with this meat substitute [109]. Reviews on the acceptance of cultured meat from the well-educated group of respondents were contrasting. The study constituted three parts: firstly, an interview with 817 people (chiefly scientists and students); secondly, an internet survey of 865 French people with well-educated backgrounds and 208 (scientists) who participated in a forum related to artificial meat. Despite the knowledge, only a scant percentage (5–11%) recommended the consumption of in vitro meat instead of farm-harvested meat. While 38 to 47% offered to support research activities on artificial meat, ironically, they speculated that the cultured meat would not be accepted as a substitute by meat-likers in the future apart from the respondents who already favored cultured meat. As a conclusive remark, the educated people underpinned the acceptance of cultured meat for environmentally friendliness and a customized nutrient profile but were not convinced with the taste and flavor of the original [110].

It is clear from the plethora of reviews that the concept of cultured meat initially revolved around the academic community involving social activists, animal lovers, animal rights activists [104], research institutes, universities, and laboratories primarily involved in the domain of meat culture [111]; however, after the debut of the lab-grown-meat-made hamburger in 2013, several entrepreneurs, start-ups, and venture capitalists have transformed it to the commercial level. Strengthening the community is strongly influenced by the exponentially increasing vegan consumers from rich countries, which was published in the World Economic Forum whitepaper “Meat: The Future Series” [112].

## 11. Technical Challenges in Production of Cultured Meat

Cultured meat production is an active commercial field of cellular agriculture using tissue-engineering technology. Production of this meat is bound with technological challenges in the raw materials used in production (e.g., bioreactor, culture medium, cell types, etc.) and the specific methods adopted for the mass production. Several start-ups and reputed forms have begun to invest and also to resolve the persisting issues in this field. The protocol for commercial production largely depends upon the choice of cell types and medium. In case of the bovine satellite cells, the optimization of a micro-carrier or cell-aggregate base is a good choice. Experimental trials suggested that packed bed reactors are reported to offer the highest efficiency due to having proportionally equal cell density. However, the chief constraint in the up-scaling process is the cost effectiveness [113]. The standard 2D technique that is currently used needs to be advanced with the integration of techniques such as 3DP as discussed earlier for better texture and appealing nature. Conventionally, growing the cells in the bioreactors is carried out for mass production, despite the surface availability, the growth of the cells is checked and maintenance under optimum control is now possible. This is often hampered due to the cell aggregation, which leads to inadequate breathability of the cells. This can be resolved through uniform concentration, pH, temperature, and other physico-chemical factors. Overall, the production is dependent upon the four key factors, viz., the count of the cells in the culture, the time period required for the complete culture, the output/yield per batch of culture, and the quantity of medium required for the same. However, this is a trial-in-trial process, and optimization is required to standardize the culture. To augment these conditions, the Cultivated Meat Modelling Consortium (U.S.) aimed to ease and resolve these interfaces through computer-based modelling of cell culture technology. Zhang et al. [114] summarized that the potential solutions for in vitro meat culture that address the issues of bioreactor engineering, nutritional science, the role of material science for preparation of cell, standardizing culture protocols, and the augmentation of cost-effective media. On the whole, the science of cultured meat is intrinsic and complex, and it is indispensable to boost the interdisciplinary approach such as bioreactor engineering and synthetic biology for the future prospects and scale-up processes [114]. One of the prominent cost drivers in in vitro meat production is the culture media, which are often produced from animal-derived sources (fetal bovine serum); however, potential research on alternative production strategies is required for promoting cost effectiveness [115].

Bringing the exact match to traditional meat texture is a tough task; more intricately developing the vascular system, connective tissues along with endo-, peri-, and epimysium of the muscle and fat layer is far more technically challenging. However, the co-culture of varied cells such as myoblasts with fibroblasts and adipocytes [116] can bring about the close match, but the problem is that each cell line is unique and requires specific media for growth and differentiation. Overlooking these condition results in sub-optimal growth, which directly reflects on the texture and taste of the in vitro meat [117].

## 12. Sensorial and Nutritional Aspects of In Vitro Meat

The question whether cultured meat is biologically equivalent to traditional meat continues to be a debate. Comparative studies based on molecular and sensorial characteristics have been reported from the point of tissue-engineering and meat technology. It is truly challenging for cultured meat to match traditional meat in terms of the nutritional components such as proteins, fats, vitamins, minerals, and other important nutrients [118]. It is noteworthy that many of the biochemical metabolites that are present in the conventional meat are net products of the food intake and the biological metabolism but are not derived from the muscle itself. These can be roughly matched up by the formulation of specific medium, and the crucial point relies on the how best the cells uptake these nutrients from the medium and grows eventually. Failure of uptake significantly reflects on the taste, nutritional, and sensorial factors of the cultured meat [119]. Post-mortem metabolism in the animal after slaughter converts the flesh into meat, and it is predominantly influenced by the anaerobic glycolysis, where the glycogen in the muscle is converted to lactate. While the role of actin and myosin contraction is due to the series of events such as calcium metabolism, the lowering of pH, thus, influences the formation of a permanent acto-myosin complex [120] resulting in “rigor mortis”. This natural biological phenomenon is, however, absent in the cultured meat, and there are no scientific studies to prove it so far. Similarly, conventional meat is subjected to tenderization, and depending on the type of meat that is used, e.g., beef, pork or chicken, etc., enzymes such as protease, calpain, proteasome, caspase, lysosomal enzyme cathepsins, and many others are involved in the tenderization and flavor enhancement [121]. Ironically, the microenvironment in the bioreactor and the intracellular conditions in cultured meat extensively vary from the traditional one. Interactions of the biomolecules obtained from the proteolysis and glycogen cycles influence the length, complexity, and water-holding capacity of the sarcomere [121].

Myoglobin is related to color and the iron content of the meat. Infusion and expression of myoglobin in culture meat is associated with media selection, sustenance of hypoxia in the reactor as well as selected media additives such as lipids or acetic acid [122]. The conventionally used media such as IMDM, RPMI1640, and DMEM contain the minimum iron content; however, supplementation of media with iron proportionally increases the iron content in the cells, but the incorporation is observed to be limited [123]. In other ways, the absorption of iron is facilitated by certain binding proteins such as transferrin, which mediates the uptake into the cell [124]. Other significant components that add to the flavor and taste in traditional meat are the fats, lactate, and inosine 5′-monophosphate; these are brought about by the Maillard reaction and lipid degradation reactions [125]. Similarly, the inclusion of fat content in cultured meat by augmenting the medium with plant-based fats and animal-derived fats at the final stage of culture process. Most commonly, protocol involves the co-culture of adipocytes with muscle cells for enhanced fat fraction in the resultant cultured meat [126]. In parallel, increased fat content can be achieved through the utilization of adipocytes derived from adipose stem cells; they have the potency to synthesize saturated and unsaturated fatty acids [127]. In general, meat hosts several minerals and vitamins such as zinc, selenium, and iron and vitamin B_12_, respectively. The inclusion of these in cultured meat is yet another intricate task, the medium is already composed of the vital minerals and it is utilized by the cells for growth and differentiation, but the quantity of the nutrients absorbed and retained by the cell is still unclear. The addition of synthetic plant-based products containing vitamin B12 at the final stage of production could enhance the nutritional quality of the product [128].

## 13. Integrated Approaches for Food Safety Monitoring in Cultured Meat

Multidisciplinary approaches and visions are necessary to carry forward the regulatory compliance and acceptance of the technology. The IoT and other state-of-art technologies offer various choices for advanced monitoring and optimization of the tissue-engineering processes, which can reduce the production costs, improve the manufacturing rate, aiding up-scaling and commercialization. Entire bioprocess of cultured meat comply with proliferation, differentiation, and maturation stages, each of which requires customized specification such as specific media, scaffolding, and bioreactors. The integration of sensors in bioreactors and imaging techniques of scaffolds will facilitate cost-effective and quality production throughout the food supply chain [129]. Table 1 provides the information on the application of sensors in tissue-engineering technology. Recent ideas on the integration of cultured meat technology with mathematical modelling and computer fluid dynamics have gained great significance. This enabled an improved understanding on the prediction of bioprocesses (cell division, growth, and differentiation) and optimization of physical variables (fluid motion, temperature, pH, pressure, heat and regulation of oxygen, carbon dioxide levels) [130].

On the other end, the integration of sensors has enabled the quality check easier at different levels and promoted quality production at every stage of the bioprocess. The sensors are categorized into three type-based contacts with the medium, viz., the invasive and non-invasive. The former is termed as an “in-line sensor” embedded into the culture medium/fluid directly and establishes contact with the growing tissue, while the later are the sensors that monitor the situation from outside (spectrophotometers), while the third type includes the “indirect (at-line) sensor”, which operates offline and is termed as a shunt or quasi sensor [131]. Electrochemical biosensors are applied to detect lactate and ammonia during the cell proliferation phase and also to check the toxicity levels due to an excess of ammonia production in mammalian cell lines [132]. To put it in a nutshell, the installation of biosensors for monitoring metabolites, nutrients, glucose, enzymes, and by-products would allow for potential recycling and energy conservation. While the most astonishing innovation is the multiple sensing through microfluidics that enabled the recognition of multiple biochemical analytes simultaneously [133].

## 14. Conclusions

The novel idea of cultured meat has become a valid choice for human food. It has become increasingly popular, and in other words, it has become the need of the hour to reduce greenhouse gas emissions through livestock rearing, which will inevitability reduce environmental pollution [134]. In this final part, we emphasize the regulatory directions posed by several international agencies such as the FAO, WHO, OIE, and the World Bank, which insist on the preparedness of food and survival. In case of future pandemics, there will be an increasing demand for animal protein sources in future [135]. In such an emergency crunch, in vitro meat would be the best way to curtail the food crisis and the right choice to increase the nutrient profile of the human population through protein integrated manufacturing protocols. The review deepened our understanding towards the association of meat consumption with planetary health. Recent qualitative research [136] unveiled that factors such as willingness for dietary change and awareness on global health are elevated facts from consumers motivated by environmental concerns, while, in a few cases, this is hindered by certain taboos, beliefs, and personal behavior. Ironically, the acceptance of plant-based meat is higher [137] than cultured meat among vegans, as this is another simple reason to deny real meat. However, educating ourselves about global health and personal culinary choices is most impactful way to govern the environment sustainability.

## Figures and Tables

**Table 1:** Sensors adopted for monitoring the physio-chemical conditions in the tissue engineering [129].

S. No	Sensor Type		Specifications
**Temperature sensors**			
1.	Platinum	Resistance Sensors	−200 to 1000 °C
2.	Nickel		−60 to 300 °C
3.	TSic		+10 to +90 °C
4.	IST, Rosemount ™	Thermocouple	−40 to 750 °C
5.	Krohne		−40 to 600 °C
6.	Pyroscience, Burns		0 to 50 °C
**pH sensors**			
1.	Optical—Pyroscience	pH Sensor Spots	Different ranges available (4–6; 5–7; 6–8; 7–9; total scale)
2.		pH Flow-Through Cell	
3.		pH Sensor Cap for Underwater Devices	
4.	Optical—PreSens Sensors	Self-adhesive pH Sensor Spots SP-LG1-SA	4.5–7
5.		Single-Use pH Flow-Through Cell FTC-SU-HP5-S	5.5–8.5
6.		Profiling pH Microsensor PM-HP5	5.5–8.5
7.	Electrochemical	Bioreactor pH Probe	Total scale Accuracy: ±0.1
**Oxygen sensors**			
1.	Optical—Mettler Toledo	Optical Dissolved Oxygen Sensors	8 ppb to 25 ppm with accuracy ±1%
2.	Optical—PreSens Oxygen Sensors	Self-adhesive Oxygen Sensor Spot SP-PSt3-SA	0–100% O_2_ Dissolved O_2_: 0–45 mg/L Accuracy ±0.4% O_2_ at 20.9% O_2_
**Carbon dioxide sensors**			
1.	Optical PreSens CO_2_ Sensors	CO_2_ Microsensor IMP-CDM1	range: 0.04–5% CO_2_ accuracy: ±0.01% at 0.1% CO_2_, ±0.1% at 1% CO_2_
2.	Potentiometric CO_2_ Sensor	CO_2_ Sensor InPro5000i/12/120	range: 0.145–14.5 psig pCO_2_ accuracy: ±10

## Data Availability

The data presented in this study are available on request from the corresponding author.
